# Post-translational modifications in rosacea

**DOI:** 10.3389/fimmu.2026.1931025

**Published:** 2026-10-09

**Authors:** Xuting Zhang, Hang Su, Wanying Deng, Fanghua Liu, Shougang Liu

**Affiliations:** 1 Panyu Hospital of Traditional Chinese Medicine, Guangzhou, Guangdong, China; 2 The Eighth Affiliated Hospital, Southern Medical University (The First People's Hospital of Shunde, Foshan), Foshan, Guangdong, China; 3 The First Affiliated Hospital of Guangdong Pharmaceutical University, Guangzhou, Guangdong, China; 4 Ganzhou Municipal Hospital, Ganzhou, Jiangxi, China; 5 Dermatology Hospital of Southern Medical University, Guangzhou, Guangdong, China

**Keywords:** biomarkers, phosphorylation, post-translational modification, rosacea, ubiquitination and acetylation

## Abstract

Rosacea is a chronic inflammatory skin disease that primarily affects the central facial region, including the cheeks, nose, chin, and forehead. Its clinical features include episodic flushing, persistent erythema, telangiectasia, papulopustular lesions, and in severe cases, phymatous changes such as rhinophyma. Rosacea substantially impairs patients’ quality of life, while current treatments focus primarily on symptom control. The pathogenesis of this disease involves abnormal immune responses, infiltration of immune cells, inflammatory reactions and angiogenesis, etc. Post-translational modifications (PTMs), particularly phosphorylation and ubiquitination, may play a role in initiating and sustaining these processes. This article reviews how PTMs such as phosphorylation and ubiquitination affect the pathogenesis of rosacea and assesses these PTM-regulated pathways as potential therapeutic targets.

## 1 Introduction

Rosacea is a chronic inflammatory skin disease that predominantly affects the central area of the face, presenting as recurrent flushing, persistent erythema, telangiectasia, papules, and pustules. In severe cases, hypertrophic changes such as rhinophyma may occur (1). This condition is more prevalent among middle-aged people, with a global prevalence of approximately 5% (2). The onset of rosacea is influenced by multiple factors, including genetic susceptibility, environmental factors, abnormal neurovascular regulation, and an imbalance in innate immune function (3). Pathologically, rosacea is characterized by chronic inflammation and vasodilation of the facial skin. Patients often report sensory abnormalities such as burning and stinging, which significantly affect their quality of life and mental health. Some patients may also experience complications, including anxiety and depression. Currently, recognized treatments for rosacea are limited and often ineffective, leading to frequent relapses. Recent studies have identified key molecular pathways and inflammatory networks in rosacea, including innate immune responses regulated by PTMs (4). Defining these molecular mechanisms is important because rosacea is prevalent, frequently relapsing, and associated with a substantial psychosocial burden PTMs. Post-translational modifications, including phosphorylation, ubiquitination can alter protein activity, stability, localization, and interactions, thereby modulating cytokine production and sustaining immune activation. Despite this potential connection, the precise role of post-translational modifications in rosacea remains unclear, hindering the development of targeted therapies. Further elucidation of these mechanisms and translational research is necessary. Accordingly, we conducted a literature review, searching extensively for articles on rosacea and its related post-translational modifications. Our goal was to identify pathways closely associated with rosacea that may have potential applications in treatment. By clarifying these biological pathways, we aim to provide effective treatment options for patients with rosacea.

## 2 Aetiology and pathogenesis of rosacea

The pathogenesis of rosacea results from the interaction between genetic predispositions and environmental factors. Evidence of familial aggregation and susceptibility in specific populations suggests the presence of genetic risk factors (5). For example, certain human leukocyte antigen subtypes and the cathelicidin antimicrobial peptide gene, which encodes the antimicrobial peptide cathelicidin, may be linked to the disease’s susceptibility (6). Environmental triggers, such as ultraviolet radiation, heat stimulation, emotional stress, spicy foods, alcohol, and microbial imbalances like Demodex mites, are known to exacerbate the condition (7). These external stimuli can trigger an inflammatory cascade and vasodilation in rosacea through post-translational modification mechanisms (e.g., phosphorylation and ubiquitination), leading to abnormal facial skin reactions.

### 2.1 Skin keratinocytes and barrier function

Epidermal keratinocytes play a pivotal role in the pathogenesis of rosacea by sensing environmental triggers and amplifying innate immune responses (**Figure 1**). Increased expression of Toll-like receptor 2 (TLR2) has been detected in keratinocytes within rosacea lesions, which may enhance cutaneous sensitivity to microbial products, ultraviolet radiation, temperature changes, and other external stimuli (8). Upon TLR2 activation, keratinocytes initiate host-defence responses by producing antimicrobial peptides and pro-inflammatory cytokines that contribute to pathogen clearance and tissue repair (9). In rosacea, however, this protective response appears to be exaggerated or dysregulated, thereby sustaining chronic cutaneous inflammation (10). One of the best-characterised pathways involves the TLR2–kallikrein 5 (KLK5)–cathelicidin axis, through which keratinocytes overproduce and aberrantly process cathelicidin antimicrobial peptide LL-37. Excessive LL-37 disrupts innate immune homeostasis, promotes inflammatory activation, and contributes to skin-barrier dysfunction; phosphorylation-dependent signalling events have been implicated in the regulation of this process (11).

**Figure 1:**
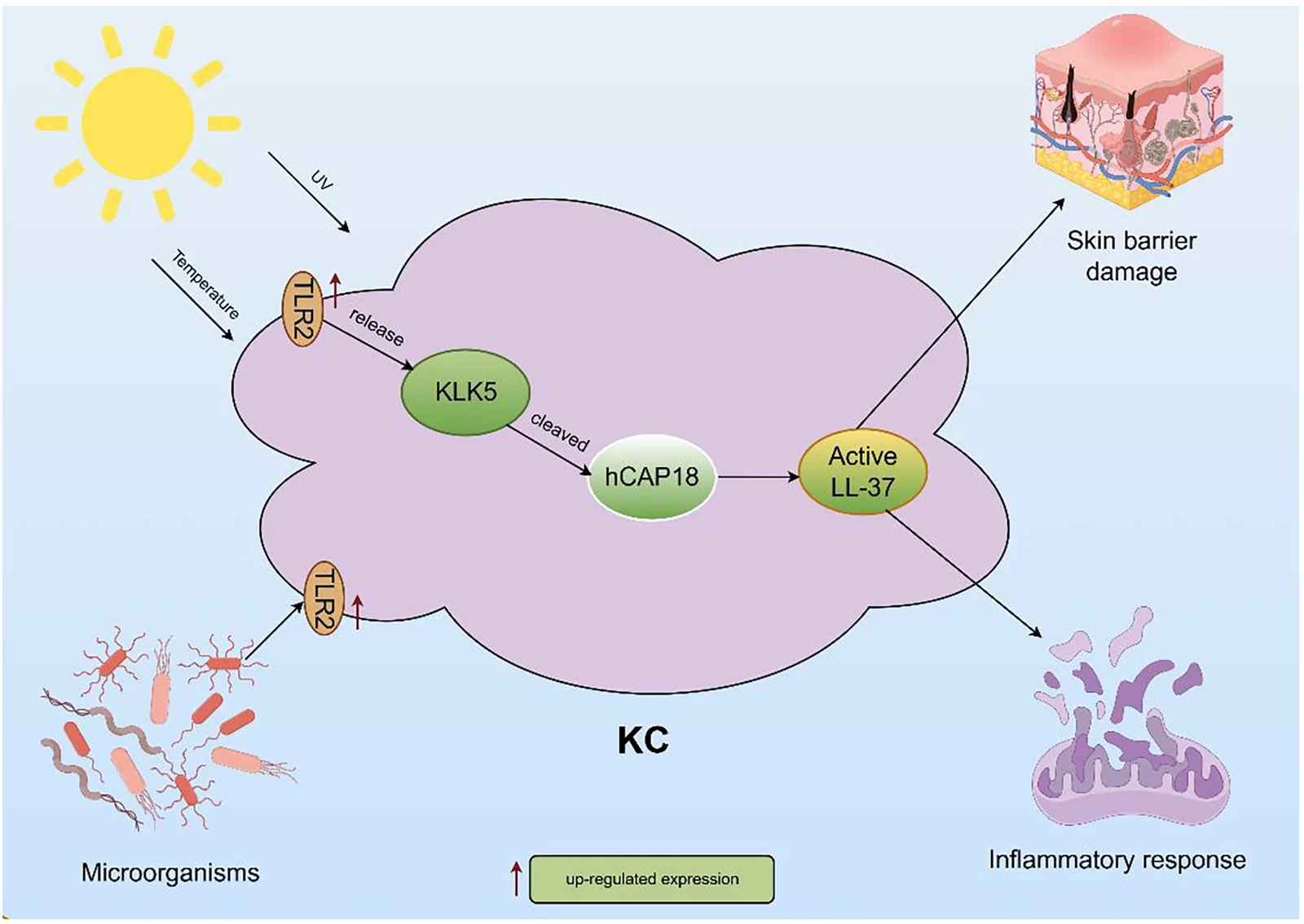
The image illustrates the TLR2-KLK5-Cathelicidin pathway in keratinocytes. The activation of TLR2 is typically induced by extracellular stressors, including ultraviolet radiation, temperature fluctuations, and microbial presence. Once activated, TLR2 facilitates the release of KLK5 by keratinocytes, thereby initiating an inflammatory response. KLK5 enzymatically cleaves hCAP18 to generate the active antimicrobial peptide fragments known as LL-37. LL-37 is notably expressed and active in rosacea, where its excessive accumulation can compromise the skin barrier and provoke an inflammatory response, thereby playing a critical role in the pathogenesis of rosacea. TLR2, Toll-like receptor 2; KLK5, kallikrein-5; KC, keratinocytes.

Recent studies further suggest that keratinocyte-driven inflammation in rosacea is tightly linked to multiple phosphorylation-dependent signalling pathways. In an LL-37-induced rosacea-like mouse model, periostin (POSTN) deficiency attenuated pathological changes, inflammatory responses, immune-cell infiltration, and angiogenesis. Consistently, POSTN knockdown in LL-37-stimulated HaCaT keratinocytes reduced the expression of TLR2, KLK5, inflammatory mediators, including tumour necrosis factor-α (TNF-α), interleukin-1β (IL-1β), and IL-6, as well as chemokines such as C-C motif chemokine ligand 2 (CCL2), C-X-C motif chemokine ligand 10 (CXCL10), C-X-C motif chemokine ligand 2 (CXCL2), and vascular endothelial growth factor (VEGF). Mechanistically, POSTN may promote inflammation and angiogenesis through activation of the Janus kinase 2/signal transducer and activator of transcription 3 (JAK2/STAT3) and nuclear factor-κB (NF-κB) phosphorylation pathways (12). Similarly, the tanshinone derivative TA20 alleviated rosacea-like skin inflammation in mice by suppressing IL-6/STAT3 signalling and reducing STAT3 phosphorylation in keratinocytes (13).

Other phosphorylation-related pathways also contribute to keratinocyte-mediated inflammatory amplification.In ocular rosacea, increased levels of phosphorylated p38 and extracellular signal-regulated kinase (ERK) have been observed, with phosphorylated proteins predominantly localised to the epidermal layer of affected eyelids, further supporting the involvement of epidermal kinase activation in rosacea-associated inflammation (14).Osteopontin has been reported to promote IL1B expression in keratinocytes through regulation of ERK1/2 and c-Jun N-terminal kinase (JNK) phosphorylation, thereby contributing to rosacea progression (15). Cinnamtannin B1 (CB1) attenuated erythema, inflammation, and neutrophil infiltration in a rosacea-like mouse model, accompanied by reduced production of myeloperoxidase and macrophage inflammatory protein-2, a functional murine homologue of IL-8. *In vitro*, CB1 reversed LL-37-induced IL-8 production in HaCaT keratinocytes and THP-1 monocytes, at least partly by downregulating ERK phosphorylation within the mitogen-activated protein kinase (MAPK) pathway (16).

### 2.2 Immune cell infiltration and inflammatory pathways in rosacea

A variety of immune cells infiltrate rosacea lesions, collectively shaping a complex inflammatory microenvironment (17). Among these cells, macrophages are particularly enriched in the dermis and are increasingly recognised as key effector cells in sustaining chronic cutaneous inflammation (18). Histological analyses of rosacea lesions have frequently demonstrated marked infiltration of macrophages and monocytes, especially around dermal blood vessels (19). Notably, macrophages in rosacea tend to acquire a classically activated M1-like phenotype (20). These M1 macrophages produce abundant pro-inflammatory mediators, including IL-1β, IL-18, and TNF-α, thereby contributing to tissue damage, immune amplification, and angiogenesis (21).

Experimental studies further support a functional role for macrophages in rosacea-like inflammation. Macrophage depletion attenuates LL-37-induced rosacea-like skin inflammation in mice, suggesting that macrophages are not merely bystander cells but active drivers of disease pathology. Transcriptomic analyses have identified guanylate-binding protein 5 (GBP5) as significantly upregulated in rosacea tissues, with its expression positively correlated with macrophage infiltration. In an LL-37-induced mouse model, GBP5 silencing alleviated rosacea-like inflammation and reduced the expression of M1 macrophage-associated genes, including IL-6, inducible nitric oxide synthase (iNOS), and TNF-α. Mechanistically, GBP5 knockdown inhibited M1 macrophage polarisation, at least partly by suppressing phosphorylation of the NF-κB p65 subunit (21). These findings indicate that GBP5 may link macrophage infiltration, inflammatory polarisation, and NF-κB-dependent cytokine production in rosacea.

Macrophage-derived signals may also influence keratinocyte inflammatory responses through intercellular crosstalk. In LL-37-stimulated HaCaT keratinocytes, LL37 could reduce the levels of phosphorylated signal transduction and activating transcription factors (pSTAT3)/STAT3 and phosphorylated AMP-activated protein kinase (pAMPK)/AMPK, while increasing the activities of phosphorylated protein kinase B/Protein Kinase B (pAKT)/AKT and mTORC1 activity. Conditioned medium derived from poly-D, L-lactic acid (PDLLA)-treated macrophages restored p-STAT3/STAT3 and p-AMPK/AMPK levels, reduced p-AKT/AKT, and inhibited mTOR phosphorylation. This macrophage-conditioned medium also attenuated downstream inflammatory events, including NF-κB nuclear translocation, VEGF production, and NOD-like receptor family pyrin domain-containing 3 (NLRP3) inflammasome-mediated IL-18 secretion. These effects were further validated in an LL-37 intradermal injection mouse model (22). Collectively, these data suggest that macrophages may modulate rosacea inflammation not only through direct cytokine production, but also through regulation of keratinocyte kinase signalling, inflammasome activation, and angiogenic responses.

Mast cells, which are widely distributed around dermal blood vessels, also play a distinctive role in rosacea pathogenesis (23). They represent an important source of LL-37 and KLK5, the serine protease responsible for cleaving cathelicidin into its active peptide form (24). Muto et al. demonstrated that mast cells are critical mediators of cathelicidin-driven inflammation, as LL-37 induces mast-cell activation and degranulation, leading to the release of histamine, chemokines, and matrix metalloproteinases (25). In mast cell-deficient mice, LL-37 injection fails to induce typical rosacea-like inflammation, further underscoring the essential contribution of mast cells to LL-37-mediated cutaneous inflammation (26).

Several phosphorylation-dependent pathways appear to regulate mast-cell activation in rosacea. LL-37-induced mast-cell activation and degranulation are accompanied by increased phosphorylation of JAK2 and STAT3, as well as elevated expression of TLR2 and myeloid differentiation primary response 88 (MyD88). GE1111, an antagonist of MAS-related G protein-coupled receptor X2/B2 (MRGPRX2/B2), inhibits the MRGPRX2-mediated mast-cell activation pathway by reducing stromal interaction molecule 1 expression and ERK1/2 phosphorylation (27). In addition, transcriptomic analysis has revealed significant enrichment of the cyclic GMP–AMP synthase/stimulator of interferon genes (cGAS/STING) pathway in rosacea lesions (28). LL-37 can induce oxidative stress-related mitochondrial damage in mast cells, resulting in mitochondrial DNA leakage. Western blotting and immunofluorescence analyses have shown increased expression of cGAS and STING, together with enhanced phosphorylation of NF-κB p65, indicating activation of the cGAS/STING/NF-κB pathway. In animal models, topical cyclosporine A or SP23 suppresses cGAS/STING/NF-κB signalling in dermal mast cells and ameliorates rosacea-like dermatitis (29). These findings suggest that mast cells may amplify rosacea inflammation through both LL-37-dependent degranulation and mitochondrial DNA-driven innate immune activation.

The inflammatory network in rosacea involves the abnormal activation and interaction of multiple innate immune signalling pathways, among which NF-κB functions as a central inflammatory hub (30). In rosacea lesions, NF-κB activation is increased and promotes the transcription of multiple inflammatory mediators, including IL-1β, IL-6, TNF-α, and CXCL8/IL-8 (12, 31). The TLR7/NF-κB/mTORC1 axis enhances the production of cytokines and chemokines, thereby facilitating the recruitment of CD4+ T cells into rosacea lesions (32). In parallel, TLR2 is a key upstream component of NF-κB activation. The TLR2/MyD88/NF-κB pathway amplifies LL-37-induced inflammatory responses and is closely linked to dysregulated innate immunity in rosacea (33). In response to inflammatory stimuli, NAD-dependent deacetylase sirtuin 7 (SIRT7) promotes TLR2 upregulation, thereby further enhancing NF-κB pathway activation (34). Thus, both the TLR2/MyD88/NF-κB and SIRT7–TLR2–NF-κB axes may represent potential therapeutic targets for controlling excessive innate immune activation in rosacea.

Additional regulatory molecules and therapeutic candidates further converge on NF-κB-related inflammatory signalling. Overexpression of DnaJ heat shock protein family member B2 (DNAJB2) alleviates rosacea-like skin damage and suppresses the expression of inflammatory cytokines, chemokines, and angiogenic mediators. DNAJB2 also inhibits endoplasmic reticulum stress marker proteins and suppresses activation of the TLR2/MyD88/NF-κB phosphorylation cascade. Moreover, DNAJB2 overexpression reduces CD4+ T-cell infiltration and downregulates genes associated with T helper 1 (Th1) and T helper 17 (Th17) polarisation (10). Quercetin-loaded cellulose nanocrystals (QL-CNCs) similarly attenuate LL-37-induced rosacea-like inflammation, reducing erythema, inflammatory infiltration, mast-cell activation, and pro-inflammatory cytokine expression, while inhibiting Th1/Th17 immune polarisation. Mechanistically, QL-CNCs enhance mitochondrial antioxidant defence by promoting superoxide dismutase 2 transport and activation, thereby facilitating reactive oxygen species clearance and suppressing NF-κB activation, as reflected by reduced p65 phosphorylation and nuclear translocation. The therapeutic effects of QL-CNCs are abolished by NF-κB activator 1, confirming the central role of this pathway (35). Minocycline also inhibits inflammatory-cell infiltration and cytokine production in rosacea-like mouse skin and cellular models. Mechanistically, minocycline blocks LL-37-induced TLR4/TNF-α activation, downregulates TLR4 and phosphorylated NF-κB, and reduces ROS production (36). Similarly, artemisinin inhibits LL-37-induced NF-κB activation and NF-κB p65 nuclear translocation in HaCaT cells (37).

Taken together, immune cells, particularly macrophages and mast cells, are central components of the inflammatory network in rosacea. Rather than acting independently, these cells interact with keratinocytes, vascular cells, and innate immune pathways to amplify cytokine release, inflammasome activation, oxidative stress, angiogenesis, and T-cell polarisation. Phosphorylation-dependent signalling, especially through the TLR2/MyD88/NF-κB axis and related ERK, JAK2/STAT3, AKT/mTOR, and cGAS/STING pathways, appears to be a key regulatory mechanism linking LL-37 stimulation to sustained inflammation. These findings suggest that targeting immune-cell activation and its associated post-translational signalling networks may provide a promising strategy for modulating chronic inflammation in rosacea.

### 2.3 Neurovascular factors and other mechanisms

Another prominent feature of rosacea is recurrent flushing and persistent vasodilation, which are thought to arise from abnormal neurovascular regulation (38). Facial skin is densely innervated by sensory nerve fibres, and these nerves appear to be hyper-responsive in rosacea. Increased expression of transient receptor potential (TRP) channels, particularly transient receptor potential vanilloid 1 (TRPV1) and transient receptor potential vanilloid 4 (TRPV4), has been observed in sensory nerves and mast cells in rosacea lesions (39, 40). Environmental and chemical stimuli, including temperature changes and capsaicin, may activate TRP channels and induce the release of neurogenic inflammatory mediators, such as calcitonin gene-related peptide (CGRP). These mediators promote vasodilation, vascular hyperreactivity, and inflammatory amplification, thereby linking sensory-nerve activation to the characteristic erythema and flushing of rosacea (41).

In addition to functional vasodilation, enhanced angiogenesis has been observed in rosacea lesions and in LL-37-induced rosacea-like mouse models. Pharmacological inhibition of angiogenesis attenuates LL-37-induced rosacea-like manifestations, suggesting that vascular remodelling is not merely a secondary consequence of inflammation but may actively contribute to disease persistence. Mechanistically, mammalian/mechanistic target of rapamycin complex 1 (mTORC1) signalling is activated in endothelial cells within rosacea lesions and LL-37-induced mouse skin. Inhibition of endothelial mTORC1 reduces angiogenesis and protects mice from rosacea-like inflammation, whereas excessive mTORC1 activation promotes vascular proliferation and aggravates rosacea-like phenotypes. Consistently, *in vitro* studies have shown that blockade of mTORC1 signalling markedly suppresses LL-37-induced tube formation in human endothelial cells. Phosphorylated ribosomal protein S6, a downstream marker of mTORC1 activation, appears to mediate this angiogenic response (42).

## 3 Post-translational modifications and rosacea

### 3.1 Phosphorylation-regulated inflammatory signalling pathways

In rosacea, immune dysregulation is characterised by the amplification of phosphorylation-dependent signalling cascades across multiple cellular compartments and inflammatory pathways (**Figure 2**). Among the post-translational modifications implicated in rosacea, phosphorylation has been the most extensively investigated. STAT3 phosphorylation is markedly increased in rosacea lesions and has been reported to correlate with interleukin-36γ (IL-36γ) expression. Inhibition of STAT3 phosphorylation attenuates inflammatory responses in both *in vitro* and *in vivo* models, suggesting that the STAT3/IL-36γ axis may represent a potential therapeutic target (43). Consistently, enhanced STAT3 activation in rosacea lesions has been associated with sustained upstream stimulation by cytokines such as IL-6 and with the induction of downstream pro-angiogenic genes (44). Single-cell RNA sequencing has further identified a distinct keratinocyte subset in rosacea lesions characterised by impaired barrier function and enrichment of interferon-γ (IFN-γ)-related signalling. Blockade of IFN-γ signalling alleviates inflammation, supporting the involvement of IFN-γ-driven phosphorylation events, particularly the JAK/STAT pathway, in rosacea pathogenesis (45).

**Figure 2:**
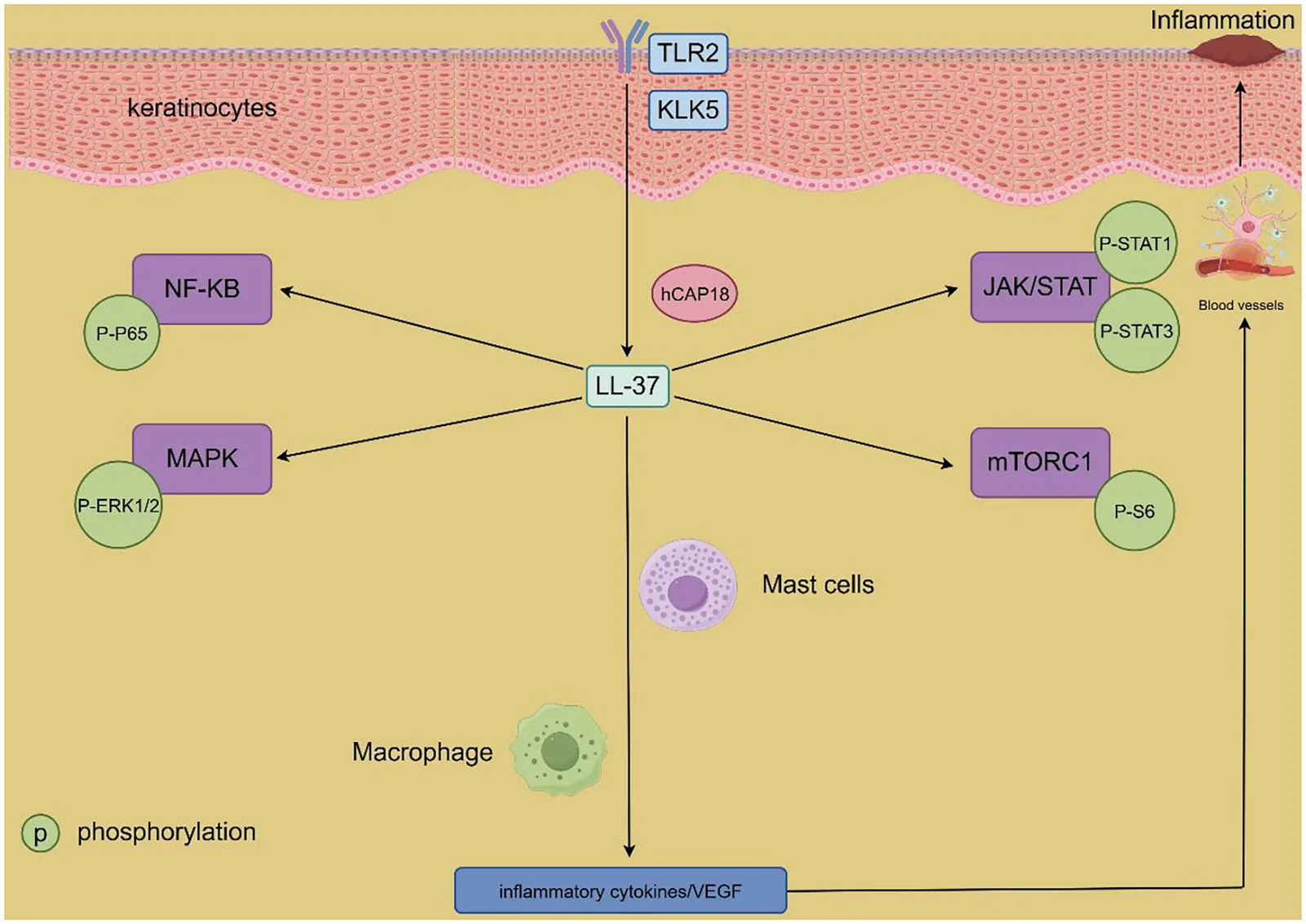
The phosphorylation signaling pathways in rosacea. When TLR2-KLK5-LL37 is activated, LL-37 can activate the JAK/STAT signaling pathway, resulting in the phosphorylation of STAT1 and STAT3. Similarly, LL-37 in the NF-KB, MAPK and mTORC1 signaling pathways, by phosphorylating the corresponding P65, ERK1/2 and S6 respectively, further stimulates downstream inflammatory factors and VEGF, ultimately leading to vascular dilation and epidermal inflammatory responses in rosacea. The phosphorylation reactions in rosacea can occur in keratinocytes, macrophages and mast cells.

Several experimental interventions further highlight the pathological relevance of JAK/STAT phosphorylation in rosacea. Cannabigerol (CBG) significantly alleviates erythema, epidermal hyperplasia, and mast-cell infiltration in an LL-37-induced rosacea-like mouse model. Topical CBG treatment suppresses the expression of cluster of differentiation 31 (CD31), vascular endothelial growth factor (VEGF), Yes-associated protein (YAP), and transcriptional coactivator with PDZ-binding motif (TAZ), while reducing activation of the JAK/STAT pathway, including decreased levels of JAK1, STAT3, and phosphorylated STAT3 (11). In keratinocytes, LL-37 stimulation markedly increases phosphorylated STAT1 levels (46). Imperatorin disrupts the interaction between JNK1 and STAT1 in LL-37-stimulated HaCaT cells, thereby suppressing STAT1 phosphorylation, nuclear translocation, and transcriptional activity, ultimately reducing the production of inflammatory cytokines and chemokines (47). Cinnamic acid also attenuates LL-37-induced rosacea-like inflammation by directly binding to STAT1, inhibiting its phosphorylation and transcriptional activation, and consequently suppressing STAT1-mediated inflammatory responses in keratinocytes (48). Collectively, these findings suggest that STAT1- and STAT3-centred phosphorylation pathways are important mediators linking LL-37 stimulation, keratinocyte activation, inflammatory cytokine production, and vascular responses in rosacea.

The mTORC1 pathway represents another key phosphorylation-dependent signalling module in rosacea. Activation of mTORC1 has been observed in the epidermis of patients with rosacea, as reflected by increased phosphorylation of ribosomal protein S6, a downstream marker of mTORC1 activity. Pharmacological inhibition or genetic blockade of mTORC1 alleviates rosacea-like inflammation in mouse models, whereas excessive mTORC1 activation aggravates disease-like manifestations. Mechanistically, mTORC1 may regulate LL-37 expression through a positive feedback loop, in which LL-37 further activates mTORC1 signalling by engaging Toll-like receptor 2 (TLR2), thereby sustaining inflammatory amplification (49). In addition, *Demodex* mites may promote activation of the phosphatidylinositol 3-kinase/protein kinase B/forkhead box protein O1 (PI3K/AKT/FOXO1) pathway, as reflected by increased phosphorylation of AKT and FOXO1. This process may enhance lipid synthesis, contribute to lipid metabolic disturbance and barrier impairment, and thereby accelerate rosacea progression (50). Metabolic alterations, including adiponectin deficiency, may further amplify abnormal mTORC1-related phosphorylation and interact with inflammatory pathways such as interleukin-17 (IL-17) signalling. The IL-17 pathway promotes inflammation and angiogenesis, whereas adiponectin supplementation partially counteracts these effects, suggesting a potential link between metabolic regulation, phosphorylation signalling, and vascular inflammation in rosacea (51, 52).

MAPK-related phosphorylation pathways also participate in rosacea-associated immune activation. In rosacea cell models, phosphorylation levels of ERK1/2 and p38 are significantly elevated compared with controls and are associated with increased expression of pro-inflammatory mediators and stress-response genes, including IL-18 (53). In mast cells, LL-37 activates MAS-related G protein-coupled receptor X2 (MRGPRX2), leading to ERK1/2 and NF-κB phosphorylation, which in turn promotes inflammatory mediator release and immune-cell chemotaxis (54). The p38 pathway may further amplify inflammation by upregulating cytokines such as IL-1 and TNF and by modulating enzymes such as inducible nitric oxide synthase, thereby increasing the production of nitric oxide and other inflammatory mediators (55).

Importantly, phosphorylation is not uniformly pro-inflammatory; it is also constrained by endogenous negative feedback mechanisms. Under sustained stimulation, phosphatases such as mitogen-activated protein kinase phosphatase 1 can be induced to dephosphorylate and inactivate p38 and JNK, thereby limiting the duration and intensity of inflammatory signalling (56). This regulatory feedback may partly explain why inflammatory responses can subside after a single transient trigger. However, in rosacea, repeated exposure to external stimuli, persistent LL-37-driven innate immune activation, microbial or mite-related triggers, and possible defects in negative regulatory mechanisms may collectively maintain cells in a state of aberrantly high phosphorylation activity. As a result, inflammatory signalling becomes recurrent, self-amplifying, and difficult to fully suppress. Overall, phosphorylation-dependent pathways, including JAK/STAT, PI3K/AKT/mTORC1, MAPK, and NF-κB signalling, form an interconnected regulatory network in rosacea.

### 3.2 Ubiquitination-mediated inflammatory regulatory mechanisms

Ubiquitination is increasingly recognised as an important post-translational regulatory mechanism in the immunopathology of rosacea. By controlling protein stability, degradation, intracellular trafficking, and signal-complex assembly, ubiquitination can both terminate excessive inflammatory signalling and provide molecular scaffolds that facilitate downstream immune activation. Dysregulation of this process may therefore contribute to the persistence and amplification of inflammatory responses in rosacea.

Huang et al. identified lysosome-associated multispanning membrane protein 5 (LAPTM5) as a macrophage-enriched molecule that is overexpressed in rosacea-like inflammation. Mechanistically, LAPTM5 binds to stimulator of interferon genes (STING) and suppresses its lysine 48- and lysine 63-linked ubiquitination, thereby impairing normal ubiquitin-dependent regulation of STING. This leads to abnormal stabilisation and sustained activation of STING (18). Activated STING subsequently engages downstream TANK-binding kinase 1 (TBK1), promoting phosphorylation of interferon regulatory factor 3 (IRF3) and activation of NF-κB-related inflammatory transcriptional programmes. These events enhance the production of type I interferons and pro-inflammatory mediators (57). In an LL-37-induced rosacea-like mouse model, either LAPTM5 knockdown or pharmacological inhibition of STING markedly attenuated skin inflammation, supporting the functional relevance of the LAPTM5–STING axis in amplifying rosacea-associated immune responses (18). These findings suggest that abnormal ubiquitin editing of innate immune sensors, particularly STING, may be an important mechanism by which macrophages sustain inflammatory activation in rosacea (**Figure 3**).

**Figure 3:**
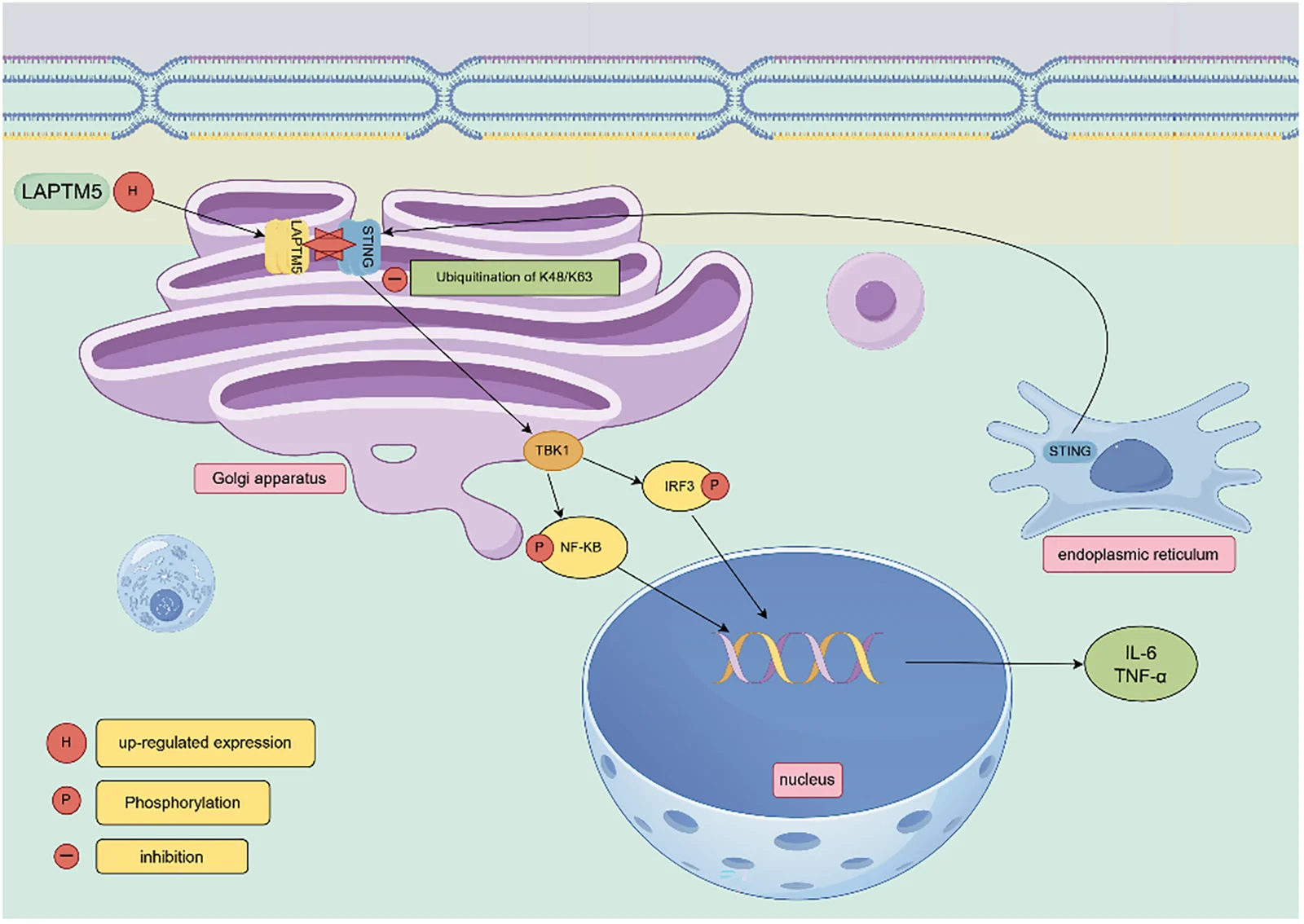
The LAPTM5-/STING ubiquitination axis in rosacea. The continuously accumulated LAPTM5 protein in macrophages can bind to STING, inhibiting the ubiquitination of STING's K48 and K63 chains, thereby making the STING protein abnormally stable and continuously activated. STING recruits the downstream TBK1 kinase and phosphorylates IRF3 and NF-κB, and simultaneously promotes the translocation of NF-κB into the nucleus, promoting the massive production of type I interferons and inflammatory factors.

Ubiquitination may also regulate keratinocyte-driven inflammation. Keratin 6A has been reported to activate STAT3 signalling and enhance the expression of pro-inflammatory cytokines in keratinocytes by reducing JAK1 ubiquitination (58). This observation indicates that altered ubiquitination is not limited to macrophage-mediated innate immune activation but may also influence cytokine signalling within epidermal keratinocytes. By stabilising upstream kinases or innate immune adaptors, impaired ubiquitination may prolong activation of pathways such as JAK/STAT, STING/TBK1/IRF3, and NF-κB, thereby contributing to persistent inflammatory signalling.

### 3.3 Other possible post-translation modifications

Other post-translational modifications in rosacea remain less extensively studied but may provide additional insight into disease mechanisms and biomarker development. Glycosylation alterations have been reported in biological fluids from patients with ocular rosacea. In a comparative analysis of tears and saliva from healthy controls and patients with ocular rosacea, sulfated glycan chains were significantly increased in both tear and saliva samples from rosacea patients. Notably, the glycan profiles detected in tears and saliva showed a high degree of similarity, suggesting that ocular rosacea may be associated with systemic or mucosal glycosylation changes. Compared with the predominance of phosphorylated N-glycans in control samples, sulfated O-glycans were enriched in samples from patients with ocular rosacea, indicating their potential value as objective biomarkers for disease diagnosis or stratification (59).

Palmitoylation may also participate in the inflammatory and fibrotic remodelling of rosacea. During the inflammation-dominant stage of rosacea, macrophage-to-myofibroblast transition (MMT) becomes evident, whereby macrophages gradually acquire myofibroblast-like features and contribute to fibrotic tissue remodelling. Macrophage depletion markedly alleviates LL-37-induced fibrotic remodelling, highlighting the pathogenic role of macrophage plasticity and MMT in rosacea. Bruceine A, a natural bioactive compound, suppresses fibrotic remodelling *in vivo* by reducing MMT and attenuating keratinocyte-driven inflammatory responses. Mechanistically, Bruceine A directly targets STAT3 and interferes with its palmitoylation-dependent activation, thereby disrupting downstream pro-fibrotic and inflammatory signalling pathways (60). These findings suggest that, beyond phosphorylation and ubiquitination, glycosylation and palmitoylation may represent emerging regulatory layers in rosacea pathogenesis, linking mucosal biomarkers, macrophage plasticity, keratinocyte–macrophage crosstalk, and tissue remodelling. The principal PTMs implicated in rosacea, together with their cellular contexts, molecular targets, current levels of evidence, and potential therapeutic implications, are summarized in **Table 1**. As summarized in **Table 1**, phosphorylation currently has the strongest mechanistic and preliminary clinical support, whereas evidence for ubiquitination, glycosylation, palmitoylation, and other PTMs remains predominantly preclinical or observational.

**Table 1:** Summary of post-translational modifications implicated in rosacea: cellular context, molecular targets, and evidence levels.

PTM type	Major cell type or Secretion involved	Target molecule/Pathway	Animal and cell experiments	Clinical studies
Phosphorylation	Keratinocytes	STAT1/STAT3, NF-κB, MAPK, and mTORC1 signalling pathways	Animal and cell experiments	JAK inhibitors, including upadacitinib (real-world study, case report), abrocitinib (case report), and Ruxolitinib (case report).
Phosphorylation	Macrophages	STAT3, NF-κB, MAPK, and mTORC1 signalling pathways	Animal and cell experiments	
Phosphorylation	Mast cells	STAT3, NF-κB, and MAPK signalling pathways	Animal and cell experiments	
Ubiquitination	Macrophages	K48- and K63-linked ubiquitination of STING	Cell experiments	None reported
Ubiquitination	Keratinocytes	Ubiquitination of JAK1	Cell experiments	None reported
Glycosylation	Tears and saliva	Sulfated O-glycosides	None reported	Clinical observational studies
Palmitoylation	Macrophages	STAT3 palmitoylation	Cell experiments	None reported

Evidence levels reflect the highest level of rosacea-specific evidence identified.

## 4 The potential and future prospects of adjunctive therapy in the treatment of rosacea

Although PTM-related pathways provide promising mechanistic opportunities for therapeutic intervention in rosacea, clinical validation of most PTM-targeted or PTM-associated strategies remains limited. Among these pathways, JAK/STAT signalling has attracted particular attention because it is directly regulated by phosphorylation and is closely linked to keratinocyte activation, cytokine production, and inflammatory amplification in rosacea. The selective JAK1 inhibitor upadacitinib has shown potential benefit in patients with papulopustular rosacea(PPR) or rosacea associated with atopic dermatitis, with a marked reduction in facial erythema reported in 10 of 12 patients (83.33%) (61). However, another study evaluating upadacitinib and abrocitinib for erythematotelangiectatic rosacea (ETR) reported heterogeneous responses, probably reflecting the small sample size (62). In addition, a case report described a 56-year-old woman with granulomatous rosacea who achieved near-complete remission after 10 months of topical treatment with ruxolitinib cream combined with metronidazole cream (63). These preliminary observations suggest that pharmacological modulation of JAK/STAT activity may be a clinically relevant approach for selected rosacea phenotypes, but the current evidence remains exploratory.

By contrast, therapeutic strategies directly targeting other PTM processes, such as ubiquitination, glycosylation, or palmitoylation, have not yet been systematically investigated in rosacea. Existing evidence linking PTMs to rosacea pathogenesis is largely derived from cell-based experiments, animal models, transcriptomic analyses, small clinical observations, or individual case reports. Therefore, the generalisability and clinical applicability of these findings remain uncertain. Future large-scale, multicentre clinical studies are needed to determine whether PTM-related pathways, particularly JAK/STAT, NF-κB, mTORC1, and STING-associated signalling, can be safely and effectively translated into therapeutic targets. Such studies should also clarify patient selection, predominant clinical phenotypes, treatment duration, safety profiles, and whether modulation of PTM-dependent signalling produces sustained clinical improvement rather than transient suppression of inflammation.

## 5 Conclusions

In conclusion, protein PTMs provide a new conceptual framework for understanding the immunopathogenesis and potential treatment of rosacea. Among the PTM-related pathways implicated in rosacea, phosphorylation-dependent signalling, particularly the JAK/STAT pathway, has been the most extensively investigated. Current evidence suggests that JAK/STAT activation participates in keratinocyte-mediated inflammation, immune-cell recruitment, cytokine production, angiogenesis, and neurovascular dysregulation. However, most mechanistic evidence remains derived from cell-based and animal studies. Preliminary clinical observations indicate that JAK inhibitors, such as upadacitinib, may improve erythema and associated symptoms, including pruritus, in some patients with rosacea. Nevertheless, the efficacy, safety, and optimal patient selection for JAK inhibition remain uncertain and require validation in larger, well-designed clinical studies.

Compared with phosphorylation, other PTMs, including ubiquitination, glycosylation, and palmitoylation, are still at an early stage of investigation in rosacea. Emerging evidence suggests that these modifications may regulate innate immune sensors, cytokine signalling, macrophage plasticity, keratinocyte activation, and tissue remodelling. Although these findings remain preliminary, they broaden the mechanistic landscape of rosacea and highlight new directions for future research. Further studies integrating molecular profiling, experimental validation, and clinical phenotyping are needed to clarify how PTM-dependent signalling networks contribute to rosacea heterogeneity and whether they can be translated into effective therapeutic targets.

## Glossary

AKT: protein kinase B
AMPK: AMP-activated protein kinase
CB1: cinnamtannin B1
CBG: cannabigerol
CCL2: C-C motif chemokine ligand 2
CD31: cluster of differentiation 31
CD4: cluster of differentiation 4
cGAS: cyclic GMP-AMP synthase
CGRP: calcitonin gene-related peptide
CXCL2: C-X-C motif chemokine ligand 2
CXCL8: C-X-C motif chemokine ligand 8
CXCL10: C-X-C motif chemokine ligand 10
DNAJB2: DnaJ heat shock protein family (Hsp40) member B2
ERK: extracellular signal-regulated kinase
ERK1/2: extracellular signal-regulated kinases 1/2
FOXO1: forkhead box protein O1
GBP5: guanylate-binding protein 5
HaCaT: human immortalized keratinocyte cell line
IFN-γ: interferon gamma
IL-1β: interleukin-1 beta
IL-6: interleukin-6
IL-8: interleukin-8
IL-17: interleukin-17
IL-18: interleukin-18
IL-36γ: interleukin-36 gamma
iNOS: inducible nitric oxide synthase
IRF3: interferon regulatory factor 3
JAK: Janus kinase
JAK1: Janus kinase 1
JAK2: Janus kinase 2
JNK: c-Jun N-terminal kinase
JNK1: c-Jun N-terminal kinase 1
KLK5: kallikrein 5
LAPTM5: lysosomal-associated transmembrane protein 5
LL-37: cathelicidin antimicrobial peptide
MAPK: mitogen-activated protein kinase
MMT: macrophage-to-myofibroblast transition
MRGPRX2: Mas-related G protein-coupled receptor X2
MRGPRB2: Mas-related G protein-coupled receptor B2
mTOR: mechanistic target of rapamycin
mTORC1: mechanistic target of rapamycin complex 1
MyD88: myeloid differentiation primary response 88
NF-κB: nuclear factor kappa B
NLRP3: NOD-like receptor family pyrin domain containing 3
p65: NF-κB p65 subunit
pAKT: phosphorylated protein kinase B
pAMPK: phosphorylated AMP-activated protein kinase
PDLLA: poly-D, L-lactic acid
PI3K: phosphatidylinositol 3-kinase
POSTN: periostin
pSTAT3: phosphorylated signal transducer and activator of transcription 3
PTMs: post-translational modifications
QL-CNCs: quercetin-loaded cellulose nanocrystals
ROS: reactive oxygen species
S6: ribosomal protein S6
SIRT7: NAD-dependent deacetylase sirtuin-7
STAT1: signal transducer and activator of transcription 1
STAT3: signal transducer and activator of transcription 3
STING: stimulator of interferon genes
TAZ: transcriptional coactivator with PDZ-binding motif
TBK1: TANK-binding kinase 1
Th1: T helper 1
Th17: T helper 17
THP-1: human monocytic cell line THP-1
TLR2: Toll-like receptor 2
TLR4: Toll-like receptor 4
TLR7: Toll-like receptor 7
TNF-α: tumor necrosis factor alpha
TNF: tumor necrosis factor
TRP: transient receptor potential
TRPV1: transient receptor potential vanilloid 1
TRPV4: transient receptor potential vanilloid 4
VEGF: vascular endothelial growth factor
YAP: Yes-associated protein
